# Cause-specific mortality patterns among hospital deaths in Tanzania, 2006-2015

**DOI:** 10.1371/journal.pone.0205833

**Published:** 2018-10-31

**Authors:** Leonard E. G. Mboera, Susan F. Rumisha, Emanuel P. Lyimo, Mercy G. Chiduo, Chacha D. Mangu, Irene R. Mremi, Claud J. Kumalija, Catherine Joachim, Coleman Kishamawe, Isolide S. Massawe, Lucas E. Matemba, Evord Kimario, Veneranda M. Bwana, Denna M. Mkwashapi

**Affiliations:** 1 National Institute for Medical Research, Headquarters, Dar es Salaam, Tanzania; 2 Sokoine University of Agriculture, College of Veterinary Medicine and Biomedical Sciences, Africa Centre of Excellence for Infectious Diseases of Humans and Animals in Eastern and Southern Africa, Morogoro, Tanzania; 3 National Institute for Medical Research, Tanga Research Centre, Tanga, Tanzania; 4 National Institute for Medical Research, Mbeya Research Centre, Mbeya, Tanzania; 5 Ministry of Health, Community Development, Gender, Elderly and Children, Dodoma, Tanzania; 6 National Institute for Medical Research, Mwanza Research Centre, Mwanza, Tanzania; 7 National Institute for Medical Research, Amani Research Centre, Muheza, Tanzania; Leibniz Institute for Prevention Research and Epidemiology BIPS, GERMANY

## Abstract

**Background:**

Understanding the causes of inpatient mortality in hospitals is important for monitoring the population health and evidence-based planning for curative and public health care. Dearth of information on causes and trends of hospital mortality in most countries of Sub-Saharan Africa has resulted to wide use of model-based estimation methods which are characterized by estimation errors. This retrospective analysis used primary data to determine the cause-specific mortality patterns among inpatient hospital deaths in Tanzania from 2006–2015.

**Materials and methods:**

The analysis was carried out from July to December 2016 and involved 39 hospitals in Tanzania. A review of hospital in-patient death registers and report forms was done to cover a period of 10 years. Information collected included demographic characteristics of the deceased and immediate underlying cause of death. Causes of death were coded using international classification of diseases (ICD)-10. Data were analysed to provide information on cause-specific, trends and distribution of death by demographic and geographical characteristics.

**Principal findings:**

A total of 247,976 deaths were captured over a 10-year period. The median age at death was 30 years, interquartile range (IQR) 1, 50. The five leading causes of death were malaria (12.75%), respiratory diseases (10.08%), HIV/AIDS (8.04%), anaemia (7.78%) and cardio-circulatory diseases (6.31%). From 2006 to 2015, there was a noted decline in the number of deaths due to malaria (by 47%), HIV/AIDS (28%) and tuberculosis (26%). However, there was an increase in number of deaths due to neonatal disorders by 128%. Malaria and anaemia killed more infants and children under 5 years while HIV/AIDS and Tuberculosis accounted for most of the deaths among adults.

**Conclusion:**

The leading causes of inpatient hospital death were malaria, respiratory diseases, HIV/AIDS, anaemia and cardio-circulatory diseases. Death among children under 5 years has shown an increasing trend. The observed trends in mortality indicates that the country is lagging behind towards attaining the global and national goals for sustainable development. The increasing pattern of respiratory diseases, cancers and septicaemia requires immediate attention of the health system.

## Introduction

Cause-specific mortality is one of the most fundamental metrics of population health. It is the most frequently used health care indicator and a valuable tool for planning and management in hospitals [1]. Most often mortality data from the National Vital Statistics System (NVSS) are the primary source of data on death events. However, the availability of such data in many low and middle-income countries (LMICs) is limited [2]. The lack of vital registration data has made countries in Sub-Saharan Africa to derive estimates of mortality using indirect demographic techniques-based and census data [3]. Despite the usefulness of population survey-based mortality data, such out-of-hospital deaths are rarely medically certified, as most of the physician-certified deaths come from hospitals [4]. The cause of death certified by a physician using the rules and procedures of the International Classification of Diseases (ICD) has been described to be the gold standard for cause-of-death reporting [5,6].

Hospital inpatient mortality is one of the quality measures that can reflect both improvements in health care and patterns in mortality over time [7]. Studies on the trends of inpatient mortality can also help researchers and policymakers assess the impact of health care quality efforts. Examining trends across patient and hospital may inform strategies for addressing disparities in health care quality by identifying groups that are leading and lagging behind in improvement [7]. Moreover, to respond effectively to changing epidemiological profiles, countries depend on reliable information on causes of mortality [8]. Monitoring mortality trends over time is also key to understanding whether or not interventions are having an impact.

There is dearth of information on causes and trends of hospital mortality in most Sub-Saharan Africa [9–11]. In the region, only few published studies in Ethiopia, Nigeria and South Africa, have focused on hospital-based mortality data [9,11–14]. However, the findings had a number of limitations, including non-adherence in the use of international coding procedures, incompleteness of data and the fact that several socio-economic and institutional factors affect the admission rate [13].

There has been little systematic data collection and reporting on the death burden due to diseases and injuries in Tanzania. Most of the available information on mortalities is based on national population-based demographic and health surveys [15–17]. In addition, data unavailability on causes of death, has resulted to all-cause or specific mortality rates estimated using statistical models which are prone to estimation error due to data incompleteness, differences in methods to ascertain causes of death and quality of health systems [18–20]. This is much more difficult for infants, neonates and deaths that occur in intensive care units [21,22]. All these emphasize on attempts that use primary mortality data. Knowing the current burden and trends of the leading causes of in-hospital death is critically important to shed light on areas that need more attention in both public health and hospital care. This retrospective analysis was carried out to determine the cause-specific mortality patterns among hospital deaths in Tanzania from 2006–2015.

## Materials and methods

### Study sites and sampling framework

This analysis involved primary (district), secondary (regional referral), tertiary (zonal referral and national) level and specialized public hospitals in Tanzania. National, tertiary and specialized hospitals were conveniently included in the study. A multistage sampling technique with a set of guiding inclusion criteria were employed to select the regional referral and district hospitals. First, all regions from the Mainland Tanzania had to be included to ensure a national geographical representation. Secondly, epidemiological variations on prevalence of malaria and HIV/AIDS [23], and population size [24] were considered to decide on the number of hospitals per region to be included in the study.

The country was divided into three strata based on population size. In highly populated regions (Dar es Salaam, Mwanza and Mbeya) three hospitals were selected from each; in medium populated (Kagera, Tabora, Morogoro, Kigoma, Dodoma and Tanga) two hospitals were selected from each region and from the lowly populated regions, one hospital was selected from each. To receive representation of primary level a 10% of all district hospitals was included. In regions where a national or tertiary hospital was included, the regional hospital was excluded.

The hospitals included in this study were one national hospital (Muhimbili), three zonal referral hospitals (Bugando Medical Centre, Kilimanjaro Christian Medical Centre and Mbeya Zonal Referral Hospital), four special hospitals (Kibong’oto Infectious Disease Hospital, Mirembe Mental Hospital, Muhimbili Orthopaedic Institute and Ocean Road Cancer Institute), 20 regional referral hospitals and 11 district hospitals. The regional hospitals were Temeke, Kagera, Kitete-Tabora, Morogoro, Maweni-Kigoma, Dodoma, Bombo-Tanga, Mara, Mount Meru-Arusha, Shinyanga, Manyara, Ruvuma, Singida, Geita, Ligula-Mtwara, Tumbi-Pwani, Rukwa, Iringa, Sokoine-Lindi and Njombe. The district hospitals included in this study were Sengerema, Ukerewe, Mpanda, Kyela, Chunya, Biharamulo, Nzega, Kilosa, Kibondo, Lushoto and Maswa.

The mechanism used to collect information on causes of inpatient death in hospitals in Tanzania is standardized for all levels of hospital. Once an admitted patient dies, the physician on call will certify the death and then use the details of the case (written in the inpatient file), confirmed diagnosis, complications that arises to establish the sequence of events to determine the immediate, probable, and underlying cause of death. This information is filled in a death register in duplicate, of which a copy is retained at hospital while the original form is submitted to the Registration Insolvency and Trusteeship Agency. This data is also filled in a death report form of which the contents are entered in an electronic District Health Information System (DHIS2) at the end of each month.

### Data collection

Pre-tested paper-based customised data collection tools were used to collect death statistics from the selected hospitals. Data collection was done from July–December 2016. A core research team comprised of public health specialists, physicians, statisticians and information system specialists was oriented on the study objectives and study tools and trained on death recording and coding of causes of deaths. Then, the core team trained research assistants who were involved in data collection. The research assistants were trained on data collection tools, research ethics and actual data collection processes. This included understanding of data sources, orientation to disease terminologies and abbreviations. Selected hospital staff were oriented and involved to support the data collection exercise. They included the medical officer in-charge, a clinician, the hospital matron or patron and at least a member of the medical records unit.

A thorough search, guided by hospital staff, of the tools (registers, report forms) used to record mortality data was conducted in all hospitals. This involved going through all mentioned storage facilities, collect and compile all identified forms and registers into a single point. The extraction process started with the source with largest number of records based on discussion with the key members of the hospital management team and review of what has been compiled. A checklist was done to mark data completeness status for that source. The next source was then taken to fill time periods where no data was found from the previous one with clear tracking of dates. This iterative process was done until all identified sources were full assessed, reviewed and all death events that occurred in the hospital were collected. Sources of data included death registers, inpatient registers and International Classification of Diseases (ICD-10) report forms. In each hospital, data collected were the deceased’s age, sex, cause and date of death.

### Data management and analysis

Data were checked for mistakes and immediate errors before entry. Quality check was done by comparing a proportion of entered data with original forms. Data entry was done using database developed in EPI Data software while STATA software (Stata Corp, College Station, Texas) was used for analysis. The causes of death tabulation was guided by ICD-10 procedures [25]. Causes of death which could not be assigned to any of the predefined categories, were grouped as “ill-defined”. Death events of which causes were not mentioned or recorded as “*unknown*” or “*undetermined*” or “*not established*” were grouped as “unspecified”. Death rates were calculated based on 2012 Tanzania Population and Census data [24]. Reported causes of death were categorized into three groups as per the Global Burden of Disease (GBD) list [26].

### Ethical considerations

This study received ethical approval from the Tanzania Medical Research Coordinating Committee of the National Institute for Medical Research (Ref. No. NIMR/HQ/R.8a/Vol. IX/2230). Permissions to access hospital data were sought and granted from the Ministry of Health, Community Development, Gender, Elderly and Children and the respective Regional Administrative Secretaries and Hospital Authorities. No one-to-one consents were sought from the relatives of the deceased patients. No individual information like names of the deceased were extracted from the sources provided, however, all entries were given identification numbers. After completion of data extraction the registers and other sources were returned to the hospital medical officer in-charge. The findings are not provided at individual level but rather in aggregated manner hence provide no link to identify individual patient data included.

## Results

### Demographic characteristics

**Fig 1:**
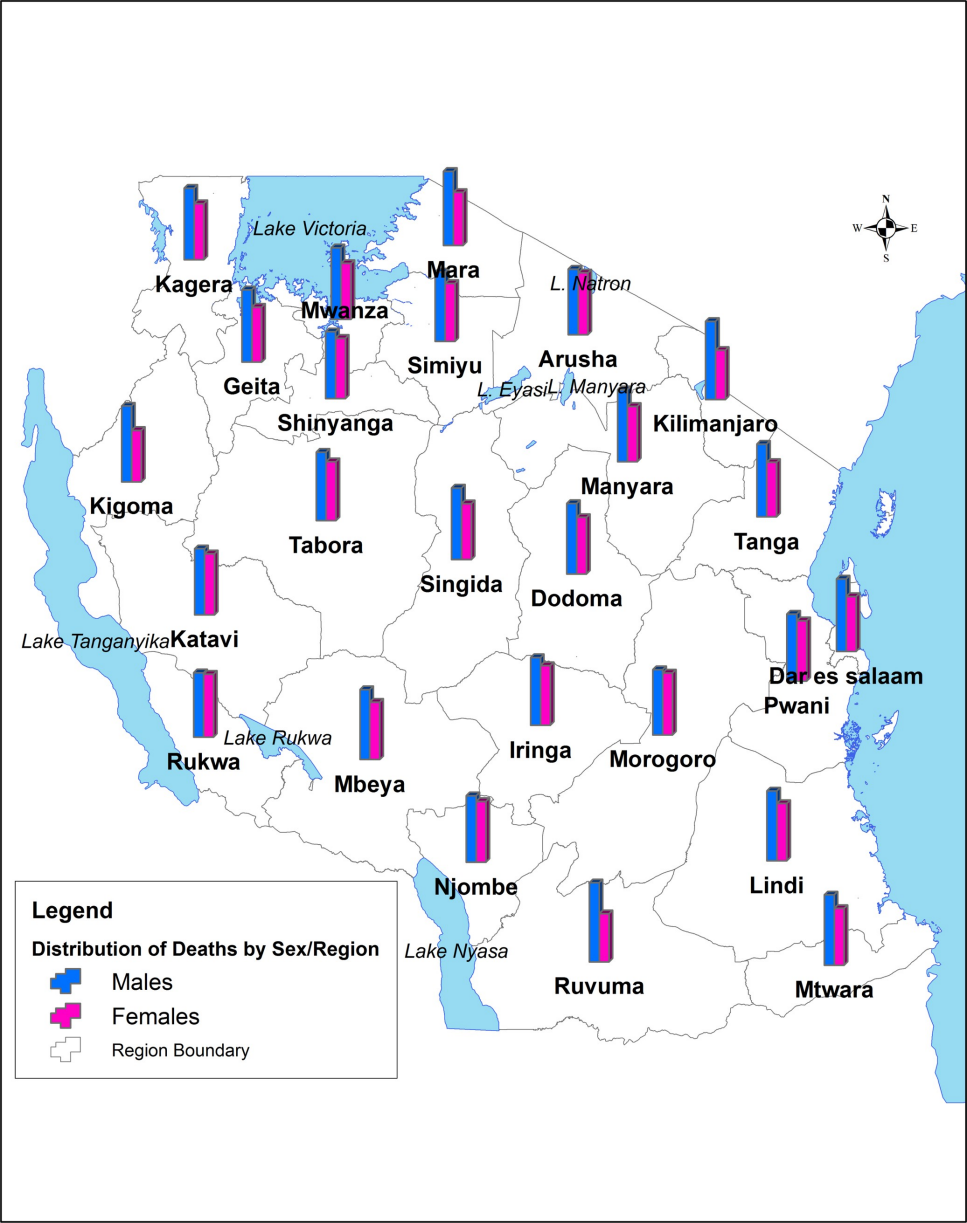
Distribution of death by sex and regions in Tanzania, 2006–2015.

A total of 247,976 deaths were captured over a 10-year period (2006–2015) in 39 hospitals. Significantly more deaths occurred among males (55.4%) than females (44.6%) (p <0.001). Region-wise, more deaths were reported in hospitals of Dar es Salaam (27.8%), Mwanza (13.3%) and Morogoro (8.7%). Regional variations in death by sex were observed. More deaths among males were observed in hospitals of Ruvuma (62.2% vs. 37.8%), Kilimanjaro (61.3% vs. 38.8%), Kigoma (59.5% vs. 40.4%), Mara (58.3% vs. 41.7%), Tanga (57.2% vs. 42.8%), Dar es Salaam (56.9% vs. 43.1%), Geita (56.8% vs. 43.2%), Singida (56.2% vs. 43.8%) and Mwanza (56.1% vs. 43.9%) regions (Fig 1).

**Fig 2:**
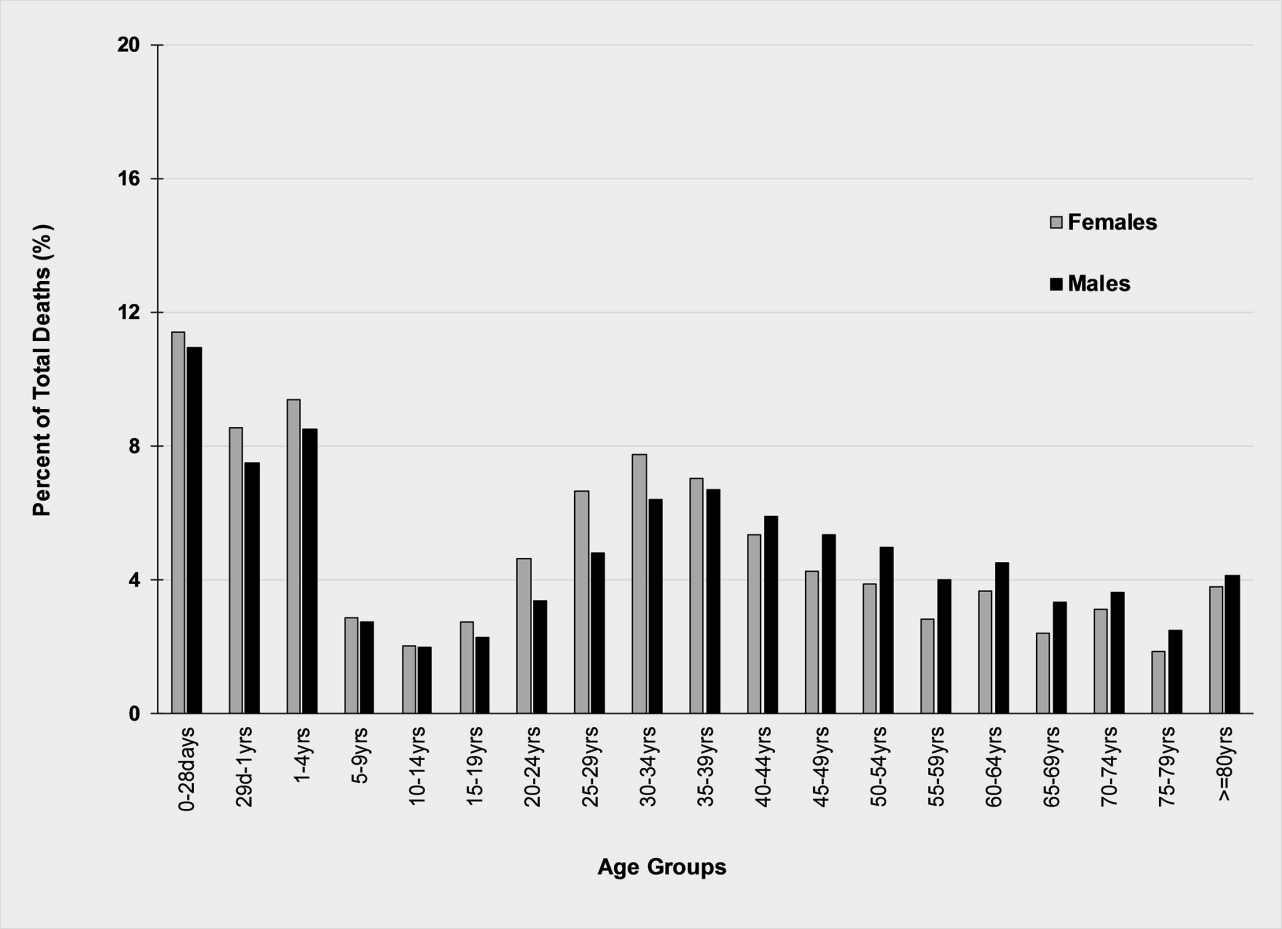
Age and sex distribution of hospital deaths, 2006–2015.

Deaths in children under five years accounted for one fifth of all recorded events. Neonates contributed to a significant proportion of deaths in this age window with the highest proportion observed among infants and children aged 1–4 years. In individuals over five years, peak of death was observed among patients 30–45 years old. The median age at death was 30 years, interquartile range (IQR) 1–50 (males = 33 years, IQR: 2–53; females = 28 years, IQR: 1–47). For age group 15–39 years, more women died than men. Beyond 39 years, the pattern changed and men accounted for the highest proportion of death (Fig 2).

**Fig 3:**
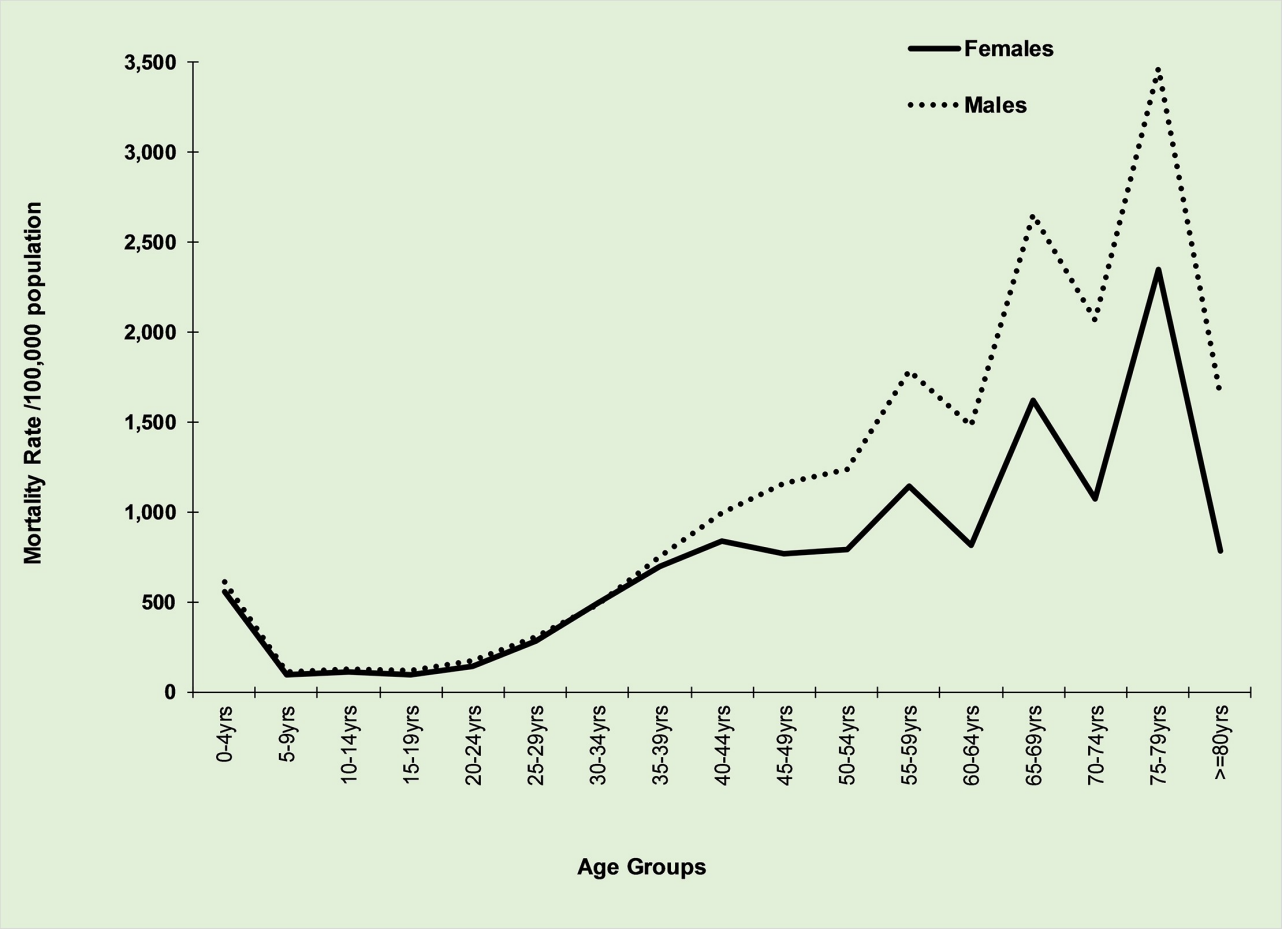
Age-sex mortality rate per 100,000 population, 2006–2015.

Age-sex mortality rate per 100,000 populations indicates a high rate among under-fives (for both males and females) and later a steady increase for individuals aged 25–29 years old and a sharp increase for those 50 years old (Fig 3). Similar rates were observed between males and females until the age category of 35–39 years. However, from age 40–44 years males indicated a departure presenting higher rates than females with a gap between the two sex groups widened as age increased (Fig 3). Mortality rate was lowest among 5–9 and 10–14 years olds. During the period of 2006–2010, higher mortality rates were reported from Morogoro, Iringa, Katavi, Mwanza and Dar es Salaam. Similarly, during the 2011–2015 period, higher mortality rates were reported from Morogoro, Iringa, Katavi, Arusha and Dar es Salaam. Exclusion of national and zonal hospitals added Mwanza among regions with the largest proportion of deaths while by excluding national, zonal and special hospitals, Dar es Salaam was not amongst those with higher death rates.

### Causes of death

**Fig 4:**
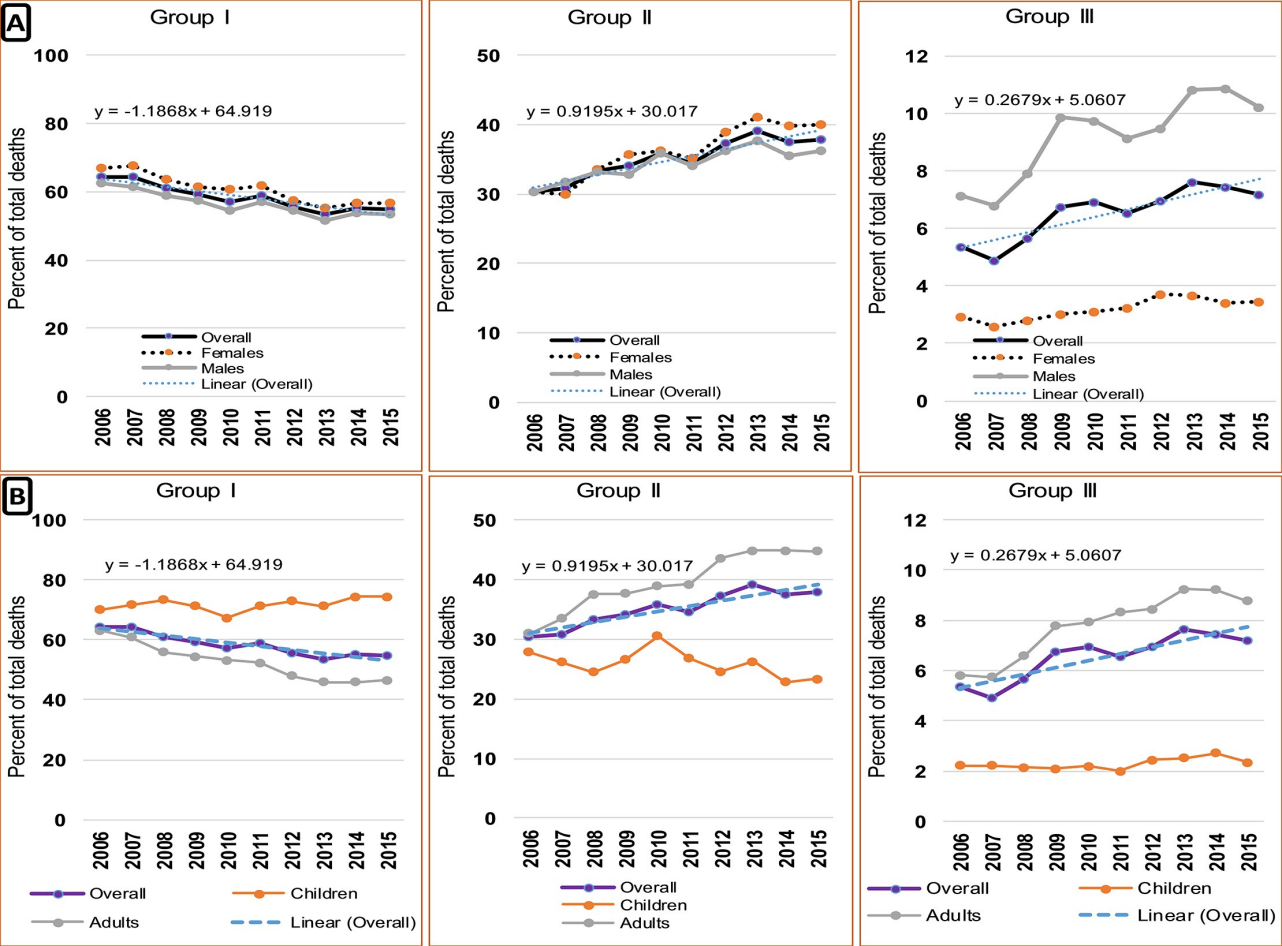
The annual pattern of causes of death by (A) sex; and (B) children and adults. The equation is for a trend-line indicating the slope and intercept over years.

The causes of deaths were categorized into Group I, II and III of the global burden of diseases. Group I included communicable diseases, maternal and perinatal causes and nutritional conditions; Group II was the non-communicable diseases while Group III included external causes of mortality. More than half (57.6%) of the deaths were due to Group I category while a third were caused by Group II. Injuries and external causes contributed to 6.7% of all deaths. A gradual decrease over the years was noted for Group I causes of death, while Group II and Group III indicate a marked increase in number of deaths for both males and females. A significant difference between males and females was observed for causes of death due to Group III, with males accounting for over twice as much the burden (Fig 4A). Group III conditions accounted for relatively higher percentage of causes of death in the regions of Rukwa, Tabora, Singida and Kigoma. Group II conditions were the significant causes of death in Ruvuma, Manyara, Dar es Salaam and Mwanza. Pwani, Geita and Katavi regions presented the highest percetage of deaths due to Group I. Spliting these patterns by age, it can be seen that for Group I conditions, children (under 5 years) presented a stagnant trend over years with a high departure from adults and the overall (all age) trend while adults showed a significant decreasing pattern. In contrary, adults were observed to account for most of deaths under Group II. On the other hand, childern presented a similar contribution throughout the 10-year period (Fig 4B). The gap between adults and children and between sex groups widened over time. A similar pattern was observed for Group III.

**Fig 5:**
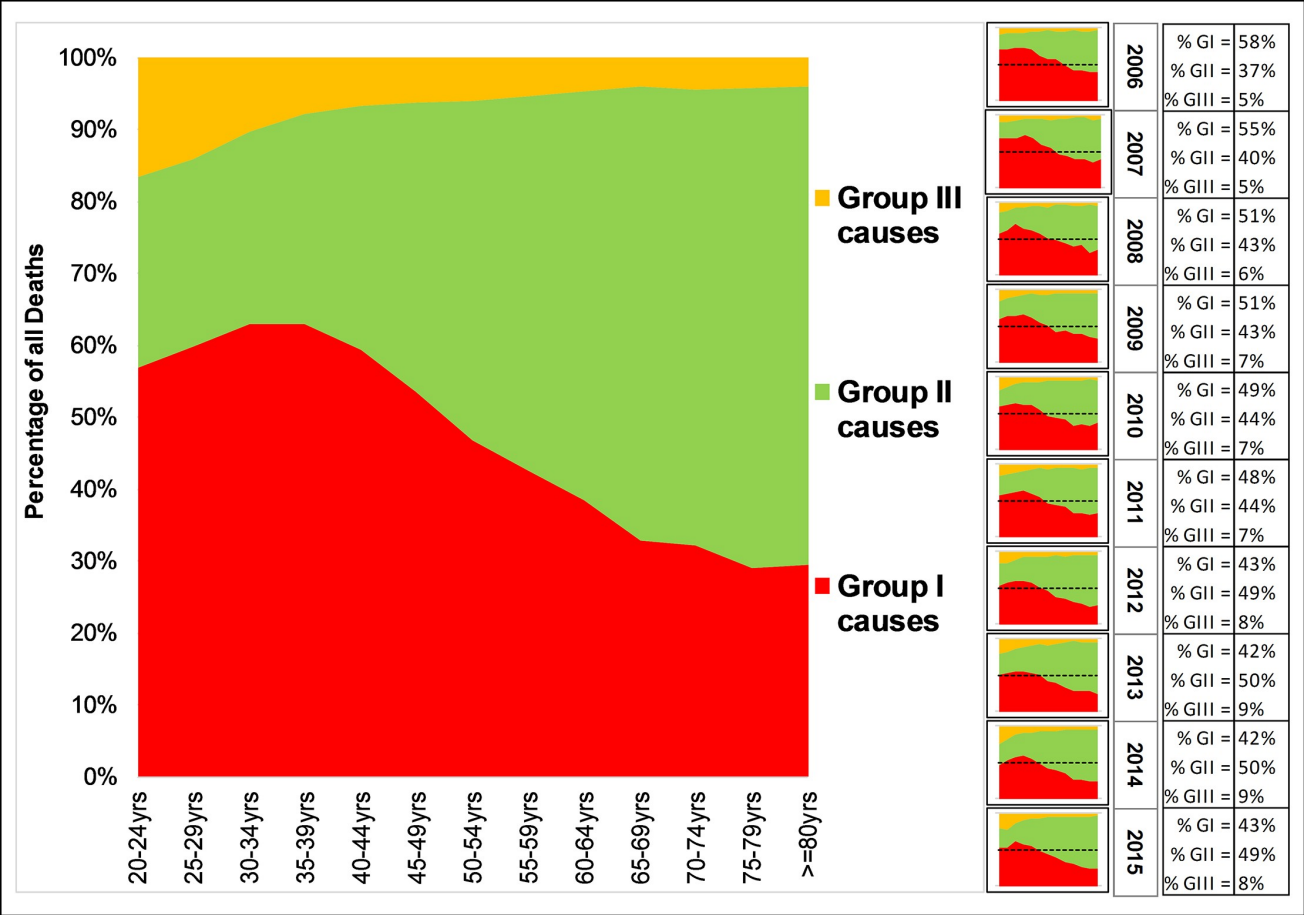
Distribution of death by the three Global Burden of diseases categories, 2006–2015. The overall contribution from young adults to old age for the entire study period is GI = 46.67; GII = 46.06 and GIII = 7.3. The annual pattern for the same age range is demonstrated on the right side of the plot, the black dotted line indicates the margin on 50% (GI: Group I; GII: Group II; GIII: Group III).

Group 1 conditions accounted for the highest proportion of deaths among children and young adults. On the other hand, Group II conditions peaked at the age of 30 years and the number of deaths increased as age increased. During the 2006–2010 period, Group I conditions accounted for about 60% of the total deaths in hospitals. The situation thereafter gradually changed to Group II conditions which accounted for 50% of the burden in 2015. The contribution of Group III to the total deaths increased from 5% in 2006 to 8% in 2015 with the individuals aged 20–34 years being most affected (Fig 5).

### Specific causes of death

**Fig 6:**
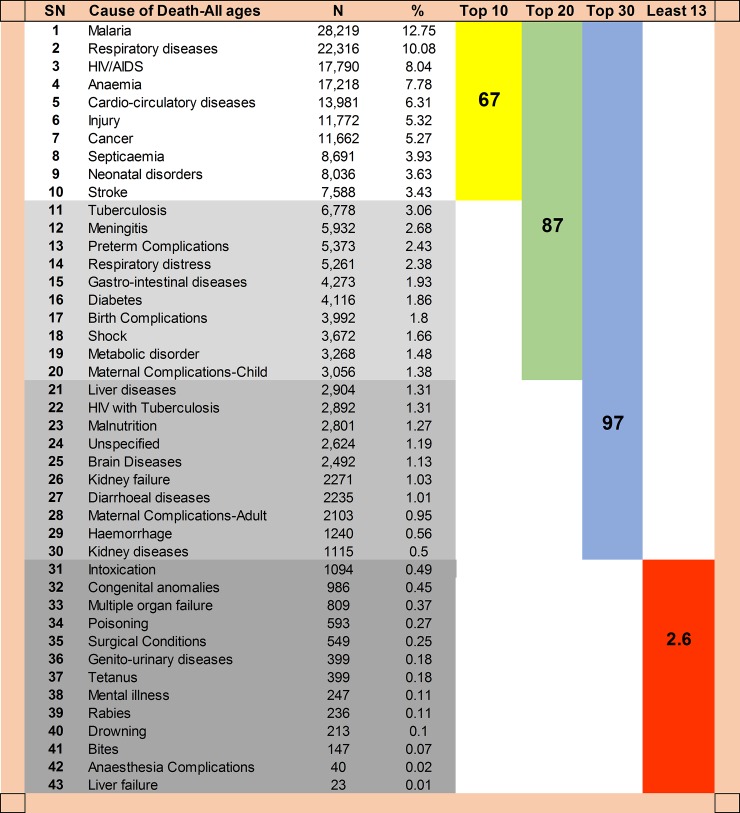
Cumulative proportion of death by cause of death groups for all ages.

Overall, the five leading causes of death were malaria (12.75%), respiratory diseases (10.08%), HIV/AIDS (8.04%), anaemia (7.78%) and cardio-circulatory diseases (6.31%) (Fig 6). About 4.2% of the recorded deaths could not fit into any of the predefined categories hence grouped as ill-defined while 1.1% of the causes were unspecified. The top 10 causes of death accounted for 67% of all deaths and included neonatal disorders and stroke.

**Fig 7:**
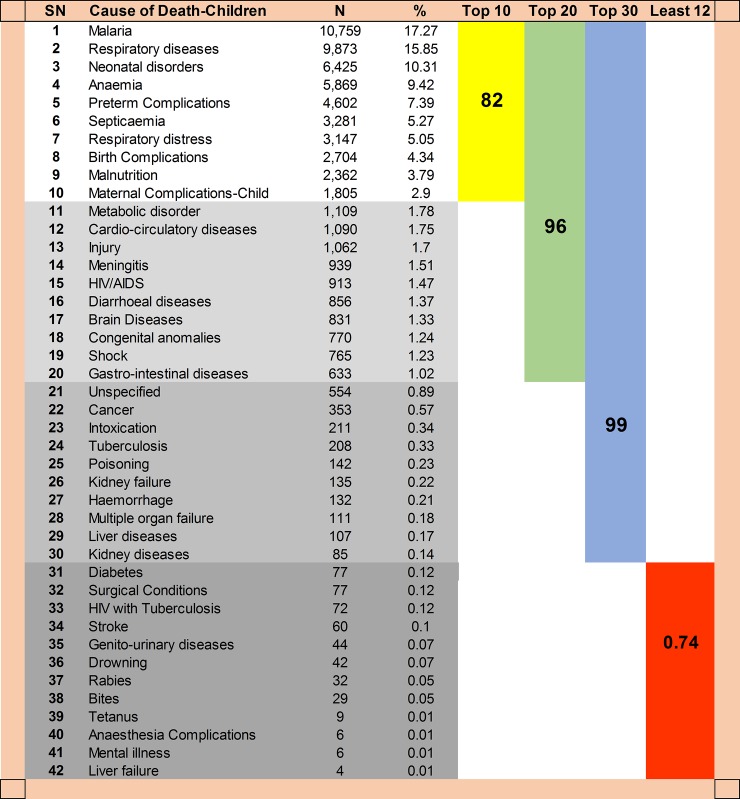
Cumulative proportion of death by cause of death groups among children < 5 years old.

This distribution changed when the deceased were categorized into children under 5 years and over 5 years old. Malaria remained the leading cause of death among children with a larger proportion (17.27%) (Fig 7) than that observed among adults (11.24%). Respiratory diseases were more profound in children (15.85%) and was ranked the 2^nd^ top cause among children and as the 4^th^ top cause among adults. Malnutrition, which ranked 23^rd^ position in the overall rating, became among the top 10 causes of death in children.

**Fig 8:**
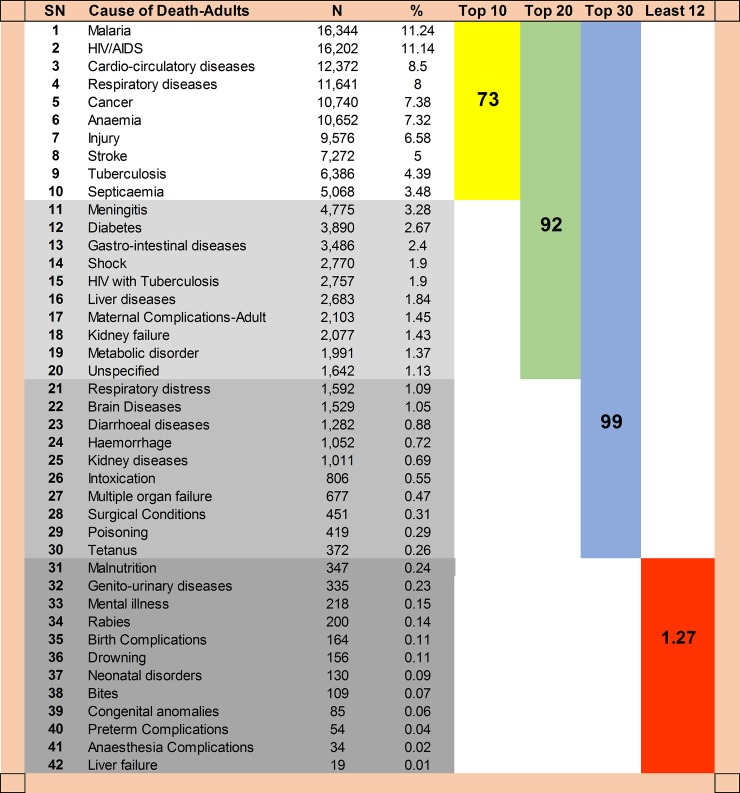
Cumulative proportion of death by cause of deaths groups among ≥ 5 years old.

Excluding the under-fives, HIV/AIDS (11.14%), cardio-circulatory diseases (8.5%), and cancers (7.38%) were ranked among the five leading causes of death (Fig 8). The top 10 causes of death accounted for 82% and 73% of all deaths in children and adults, respectively. Interestingly, the top 3 causes accounted for 43.4% in children and 30.9% in adults.

**Fig 9:**
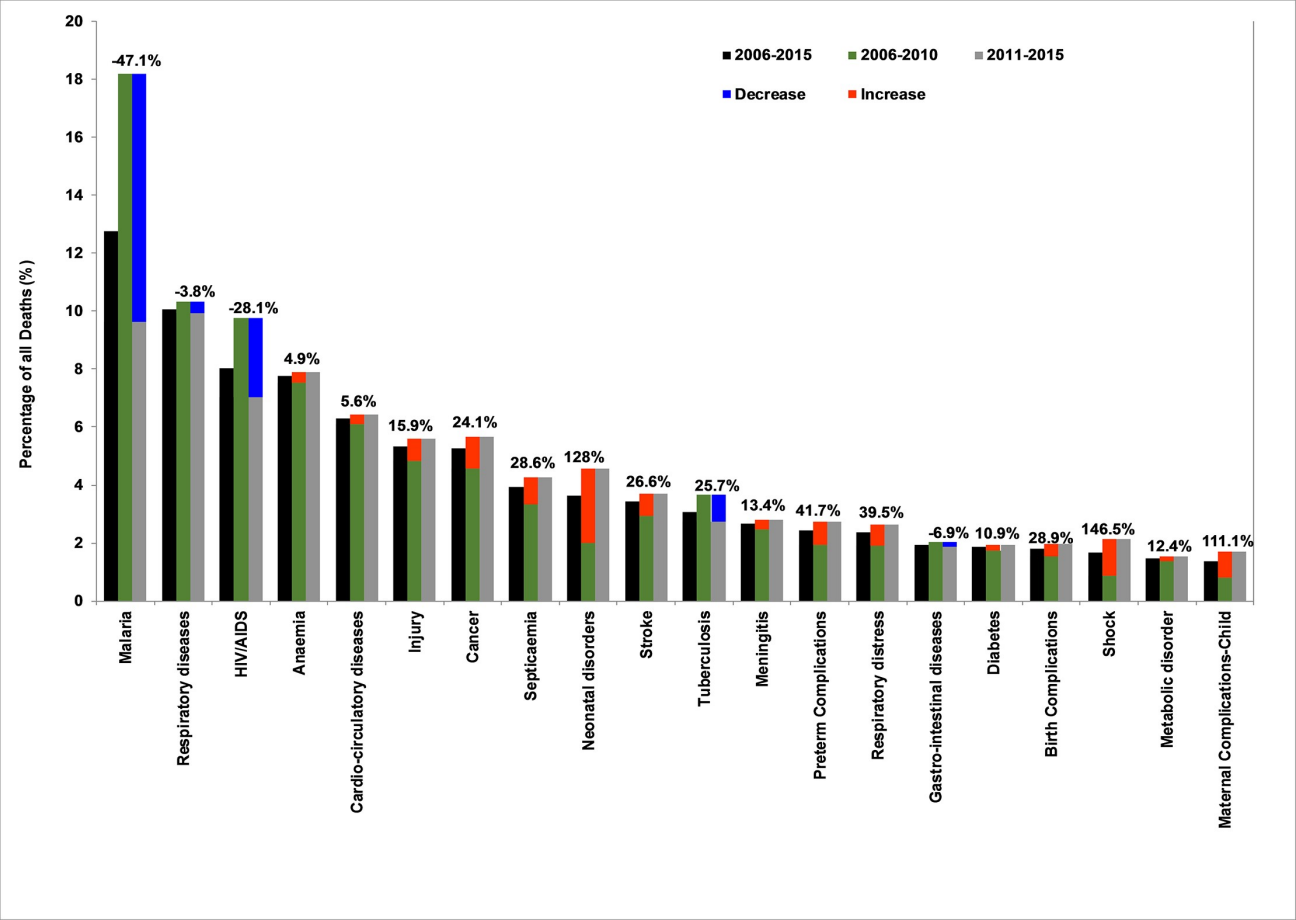
Comparing pattern of cause of death between 2006–2010 and 2011–2015 periods for all age groups. The blue bars indicate the causes with decreasing trend. Red bars indicate those with increasing trend. The actual percentage increase or decrease is shown as data label for each cause.

Comparing data between 2006–2010 and 2011–2015, malaria was the leading cause of in-hospital deaths during the two periods, followed by respiratory diseases and HIV/AIDS. However, during the 10-year period, there was a substantial decline (47%) in the number of deaths caused by malaria, followed by HIV/AIDS (28%) and tuberculosis (26%). While deaths due to malaria continuously declined significantly over the period, those due to HIV/AIDS declined during 2006–2010 period and thereafter remained almost stable. Deaths due to anaemia has remained stable over the study period, while those due to injury, cancer, strokes, septicaemia and neonatal disorders increased over the years (Fig 9). During the same period, there was an increase in the number of deaths due to shock (147%), preterm complications (42%), birth complications (29%) and child deaths associated with maternal complications (111%).

### Causes of death by hospital level

Distribution of causes of death was examined by level of hospital. Most deaths occurred at primary (district) and secondary (regional referral) level hospitals. Septicaemia was more common in secondary and tertiary hospitals, while preterm complications were mostly reported from tertiary (zonal) hospitals. Half (50%) of the neonate deaths occurred in tertiary hospitals. Infant mortalities were mostly (42%) reported from secondary level than primary or tertiary level hospitals presenting a similar pattern for children 1–4 years old. A similar trend was observed for individuals aged 5–9 years, 10–14 years and 15–19 years.

Regional referral (secondary level) hospitals accounted for the majority of deaths due to malaria, respiratory diseases, HIV/AIDS, cardio-circulatory diseases, ill-defined conditions, septicaemia, gastrointestinal diseases and diabetes. Most of deaths due to shock and pre-term complications were reported from tertiary (zonal referral) hospitals. The highest proportion of deaths due to meningitis and birth complications occurred at district hospitals whereas most of the deaths due to cancers were reported from special hospitals.

**Fig 10:**
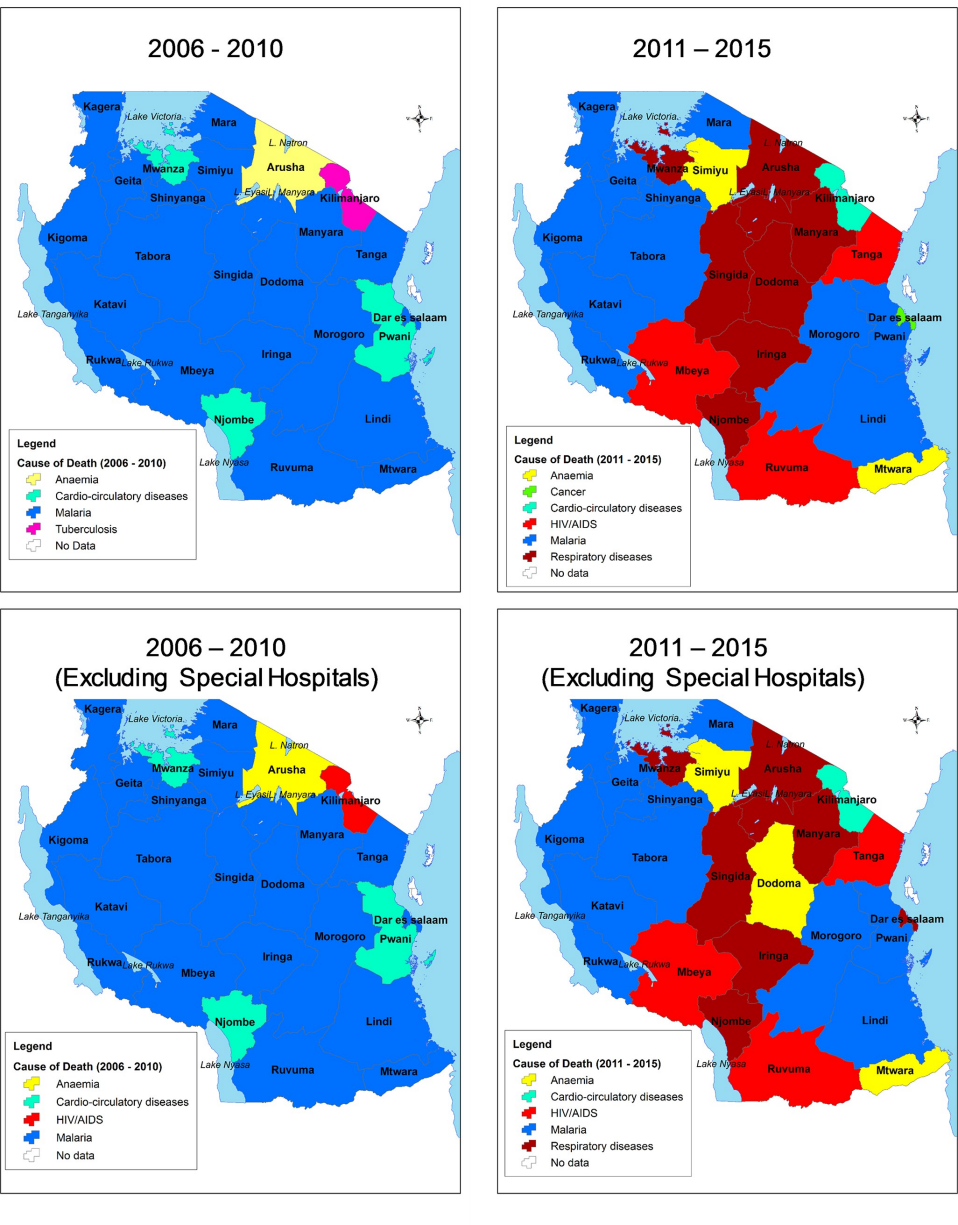
Leading causes of death in regions comparing periods of 2006–2010 and 2011–2015. Maps are shown with inclusion or exclusion of special hospitals.

Leading causes of death in each region were identified and mapped. The leading causes were compared between periods of 2006–2010 and 2011–2015 and when special hospitals were included or excluded. In the period 2006–2010 malaria was the lead cause in almost all regions with few exceptions of Arusha (anaemia), Mwanza, Pwani and Njombe (cardio-circulatory diseases), Kilimanjaro (tuberculosis when including special hospital) and HIV/AIDS (when special hospitals were excluded). During 2011–2015, hospitals in the central part of the country reported respiratory diseases as the major causes of death, with the exception of Kilimanjaro (cardio-circulatory diseases), Tanga, Mbeya and Ruvuma (HIV/AIDS). Interestingly, the regions most affected by respiratory diseases during this period (2011–2015) were those along the eastern wing of the Rift Valley. With inclusion of special hospitals, cancer was the leading cause of deaths in Dar es Salaam during the 2011–2015 period, but changed to respiratory diseases after excluding cancers (Fig 10).

## Discussion

This is the first nationwide retrospective study that analysed routinely collected hospital data to determine patterns and causes of death in Tanzania. As death is an inevitable event, its statistics should be keenly studied to provide guidance on implementation of best medical care and assess impact of health interventions. Though the methodology employed was time consuming, such attempts are necessary to establish a stable and valid baseline statistics to use for future estimation and evaluations.

The findings of this analysis indicate that more in-hospital deaths in Tanzania occur among males than females. Interestingly, more deaths occurred among children under one year of age than all other age groups. Among patients 15–39 years old, more women than men died in the study hospitals. Beyond the age of 39 years, a proportion of men who died was higher than that of women. Sex differences in mortality has been reported to vary by country [11,27–29]. Factors that influence sex differences in mortality include biological factors such as hormonal influences on physiology and behaviour, and socio-environmental factors including health-seeking behaviour and culture. Studies in Africa show that men are more likely to be admitted to hospitals than women. This is most likely to be due to the fact African men are less health conscious than women, hence most women report for treatment earlier than men. Men are reported to attend to their health when complications have set in [11].

Peak of hospital deaths was observed to occur between 30 and 45 years. The median age at death recorded in this study is similar to the findings of studies in Nigeria [11,29] and Ethiopia [14]. The median age at death in this study and in other African countries is younger than elsewhere in the world [30]. Most deaths affected neonates, infants and children under the age of 4 years while the age groups 5–19 years accounted for the lowest proportion of total deaths. Mortality rates are usually low among adolescents than other age groups. This study demonstrated an increase in the percentage of death as age increased from 15 to 39 years, followed by a decline at 79 years. This trend was similar among both males and females. Previous studies have reported a tendency of levelling off of the mortality rate at very old ages (>85)–the so-called old-age plateau [31]. However, other studies have found that the rate of mortality increase becomes either faster or slower than the predicted exponentially increasing trajectory [31–33]. Most neonate and infant deaths occurred at tertiary and secondary level hospitals, respectively.

Most of specialized hospitals presented with deaths related to their area of specialization, other causes of death were equally distributed to all levels of facilities with a very minimal variation. However the larger number of neonatal deaths in zonal referral hospitals is likely to reflect a flaw either on quality of paediatric care or timing of referral of cases from lower level hospitals. Ideally the referral hospitals are well equipped and have qualified staffs, but if decisions to refer are not timely, little can be done to save the lives. There were more deaths due to infectious diseases in the district hospitals while deaths due to neonatal, preterm, and non-communicable conditions were largely recorded in the secondary and tertiary hospitals indicating that conditions which tend to present with chronicity and complications are likely being referred and managed in the higher level facilities.

The magnitude of variability in patient outcomes between hospital levels and geographical location in Tanzania is large. Overall, higher mortality rates were reported from Morogoro, Iringa, Katavi, Mwanza and Dar es Salaam. Moreover, Morogoro and Iringa reported the highest proportion of deaths due to malaria, tuberculosis and HIV/AIDS, which are likely to have contributed to the majority of deaths in these regions. The variations in death rates between geographical locations have been reported by other studies [34]. The magnitude of mortality in hospitals varies from region to region and is more affected by state of hospitalization, duration of admission, quality of services provided, number of co-morbid conditions, and type of illness among others [35–36]. Lack of capacity and insufficient diagnostic resources and medicines have resulted to most of the deaths which occurred in hospitals [6]. Human resources capacity, skills and availability have been implicated to high morbidity mortality rates in Tanzania [37], a country that is experiencing human resource crisis and inadequate health system capacity [38–39].

Overall, majority of deaths were attributed to Group I diseases (communicable diseases, maternal conditions and nutritional deficiencies). However, over the years, there has been a significant increase in deaths caused by Group II (non-communicable diseases) and Group III (injuries). A similar trend has been reported elsewhere in Africa [12]. In 2015, statistics indicate that more than half of all deaths in low-income countries were caused by the Group I conditions. During this period, lower respiratory infections were among the leading causes of death in low-, middle-, and high income groups [35]. The high concentration of deaths from Group I conditions in Africa indicates that the region is still in the early stages of the epidemiological transition.

The leading specific causes of hospital death in Tanzania included malaria, respiratory diseases, HIV/AIDS and anaemia. Similar observations have been reported elsewhere in Africa [29,36]. Deaths due to malaria, HIV/AIDS and tuberculosis declined over the study period. The decline in deaths due to malaria is likely to be attributed to the decline in incidence and prevalence of the disease during the past decade associated with intensive interventions [37]. A similar decline followed by stabilization in the prevalence of HIV in Tanzania observed in this study has been previously reported by other workers [38]. The availability of antiretroviral drugs is described as a key factor contributing to the decline of deaths among HIV-infected patients and prolonging the lives of those infected [39], hence the observed stabilized mortality pattern. The increase in respiratory diseases and sepsis related deaths requires further studies to establish factors behind the observed patterns. With the persistently high death rate due to respiratory diseases, the country need to seriously consider putting in place a specific programme to address the threat.

Cancers and injuries ranked as 7^th^ and 8^th^ leading causes of death in hospitals of Tanzania. Globally, cancer is the second leading cause of mortality and was responsible for 8.8 million deaths in 2015 [40]. It is estimated that about 70% of deaths due to cancer occur in low- and middle-income countries. The increasing cancer incidence and its associated mortality in low-and-middle-income countries has been associated with increased prevalence of key risk factors and population aging [41]. The high residual burden of infectious agents (such as HIV/AIDS, human papillomavirus, hepatitis B virus) in some sub-Saharan African countries still drive the rates of certain cancers. Available statistics indicate that about one-third of all cancers in the region are estimated to be infection-related [42–44].

Injuries have emerged among the top-ten causes of death reported by hospitals in Tanzania. Road traffic injuries (RTIs) rank high among the major causes of death, claiming more than 1.25 million deaths each year worldwide [35]. According to recent WHO statistics the Tanzania age adjusted death rate due to RTIs is 32.9 per 100,000 of population [45]. RTIs account for the highest proportion of unintentional injuries that are increasingly recognised in low-and middle-income countries as a major cause of morbidity, permanent disability and mortality [46]. The problem is increasing at a fast rate in developing countries due to rapid motorization, weak enforcement of traffic regulations and other factors.

### Limitation of the study

Following the nature of the study design, which comprised of collection of long term retrospective data, this study was subjected to a number of challenges. These included poor record keeping, non-adherence to ICD-10 procedures, data incompleteness and unavailability of data in some hospitals. All these are likely to have been attributed to inadequate number of clinicians, inadequate training on ICD and death certification. The study however, assumes that the records obtained were accurate to the acceptable clinical level.

## Conclusion

In conclusion, the leading causes of in-hospital death in Tanzania are malaria, respiratory diseases, HIV/AIDS, anaemia and cardio-circulatory diseases. Deaths in children under five years’ account for one fifth of all inpatient hospital deaths. Peak of hospital death occur among young adults, the productive age group. Although a decline in deaths due to communicable diseases has been observed, deaths due to non-communicable diseases and injuries increased over the recent years affecting more male individuals. The observed trends in mortality indicate that the country is lagging behind towards attaining the global and national goals for sustainable development.
